# POCUS Confirmation of Intraosseous Line Placement: Visualization of Agitated Saline within the Right Heart in a Critically Ill Infant 

**DOI:** 10.24908/pocus.v8i1.16200

**Published:** 2023-04-26

**Authors:** Inbar S Y Plaut, Zachary W Binder

**Affiliations:** 1 Pediatric Emergency Department, University of Massachusetts Chan Medical School, University of Massachusetts Children’s Medical Center Worcester, MA USA

**Keywords:** IO Line, Resuscitation, Pediatrics, Cardiac POCUS

## Abstract

Intraosseous (IO) line placement can be a life-saving procedure in the management of critically ill patients. Confirmation of correct IO line placement can be difficult. Prior studies have examined the use of point of care ultrasound (POCUS) to confirm IO line placement by using power Doppler over bone to detect flow within the intraosseous space. This case illustrates a novel use of POCUS in which agitated saline is visualized within the right heart as a means of confirming correct IO placement.

## Introduction

Intraosseous (IO) line placement is a fast and effective way of infusing fluids and medications. Confirmation of correct placement and patency of IO lines can be difficult, especially in young children. An improperly placed IO line can lead to extravasation into surrounding soft tissues which can cause tissue necrosis^1^ and compartment syndrome 2. Additionally, a patient with an improperly placed IO will not receive fluids and medications as intended. Traditional methods for attempting to confirm IO line placement include the ability of the needle to stand upright without support, the ability to aspirate bone marrow, and the ability to infuse crystalloid easily without visible extravasation 3. These traditional methods lack the reliability required in a critical resuscitation. The ability to aspirate bone marrow, while revealing when present, has been shown to be present in only 68.5% of properly placed IO lines 4. The ability to determine fluid extravasation is subjective and often challenging, especially in pediatric patients. 

Prior studies have examined the use of point of care ultrasound (POCUS) to confirm IO line placement. The use of power Doppler over bone to detect flow within the intraosseous space, has been shown to be 100% sensitive and 100% specific in a small cadaver study (n = 8) 5.A larger study, utilizing a similar technique on sedated swine (n =72), has been shown to be 72% sensitive and 79% specific 6. Saul et. al. found the use of ultrasound to detect fluid extravasation into soft tissue to be 94% accurate 4. 

We describe a case in which POCUS was used to visualize agitated saline within the right heart as a way to confirm IO placement. Agitated saline augmented echocardiography is used clinically to evaluate for intracardiac shunts. Recent studies have demonstrated the ability to confirm central venous catheter (CVC) line placement in neonates through visualization of agitated saline 7. We were unable to find literature describing agitated saline augmented echocardiography to confirm IO line placement. 

## Case Report

A 4-week-old male was brought to the hospital for evaluation of diarrhea and decreased responsiveness. The patient had been discharged from the hospital 24 hours prior after a multiday admission for the evaluation of fever and diarrhea. In triage the patient was noted to be in extremis and was rushed back to the pediatric emergency department. On initial evaluation by the medical team the patient was noted to be hypothermic to 31.9 degrees C (89 degrees F), ashen, unresponsive, with irregular respirations, delayed capillary refill and was producing copious liquid stool. Cardiopulmonary resuscitation (CPR) was initiated. Initial attempts at intravenous (IV) access were unsuccessful and a right tibial IO was placed. The patient’s presentation was concerning for uncompensated hypovolemic shock and aggressive fluid resuscitation was initiated. 

**Figure 1:**
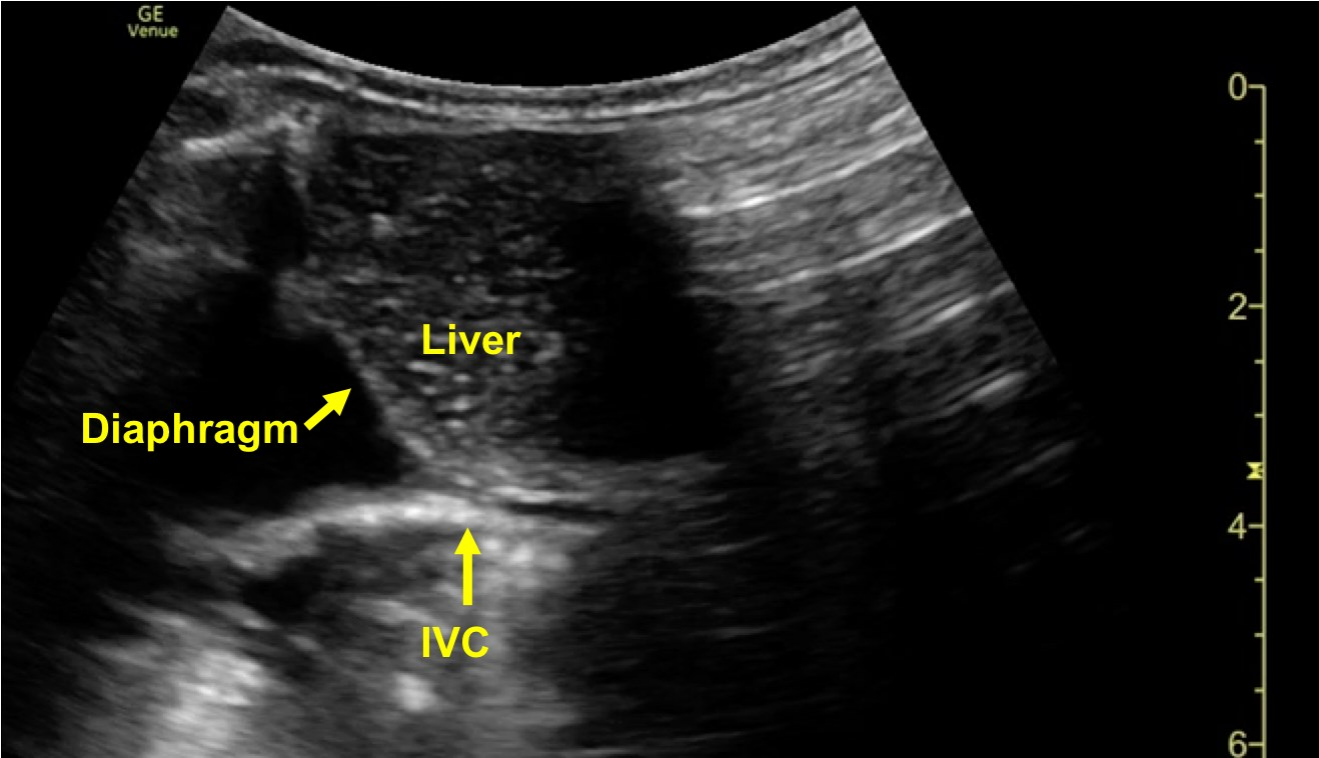
Inferior vena cava (IVC) view using a curvilinear probe in EM convention demonstrating a collapsed IVC.

**Figure 2:**
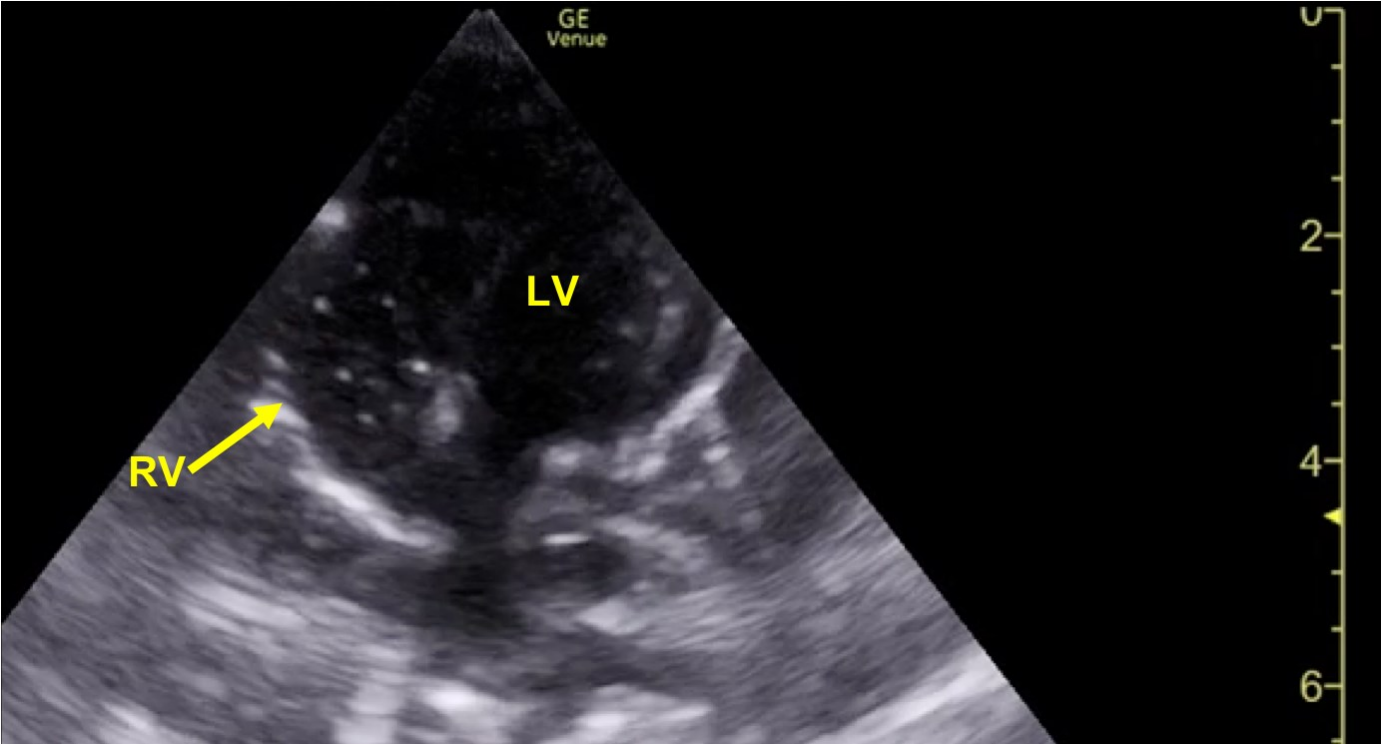
Subxiphoid short axis view of the heart demonstrating bubbles (hyperechoic circles) within the right ventricle (RV).

The diagnosis of hypovolemic shock was supported by initial laboratory values: pH <7.00, bicarbonate 3 mmol/L, lactate 6.49 mmol/L, creatinine 1.3 mg/dL (prior 0.28 g/dL), an aspartate transaminase (AST) of 80 U/L and an alanine transaminase (ALT) of 94 U/L. After a single round of CPR, central pulses were palpable with a heart rate (HR) of 88. Compressions were discontinued and a cardiac POCUS was performed. Parasternal long axis, parasternal short axis, sub-xiphoid 4 chamber, and sub-xiphoid short axis views were obtained using a phased array probe in a pediatric cardiac preset. Additionally, an inferior vena cava (IVC) view was obtained using a curvilinear probe in EM convention. The POCUS revealed a collapsed IVC (Figure 1, Supplemental Video S1), normal cardiac function, and an absence of a pericardial effusion. While performing the subxiphoid short axis evaluation, with volume resuscitation ongoing via infusion of normal saline through the IO line by means of premeasured 10 milliliter syringes, bubbles were serendipitously appreciated within the right heart (Figure 2, Supplemental Videos S2, S3). The presence of bubbles within the right heart moments after the saline was infused helped to confirm the proper placement and functioning of the patients tibial IO line. As volume resuscitation continued the patient began to exhibit a weak cry and spontaneous movements. After a prolonged hospitalization in the Pediatric Intensive Care Unit, complicated by disseminated intravascular coagulation (DIC), the patient was ultimately discharged home. During subsequent subspecialty evaluation, the diagnosis of a rare tumor necrosis factor (TNF) receptor associated factor 3 deficiency leading to a systemic inflammatory syndrome was made via genetic sequencing. 

## Discussion

This case describes a novel technique for confirming IO line placement. An advantage of our proposed agitated saline visualization technique is that cardiac POCUS is often performed during resuscitations, and no additional ultrasound views are required. The subxiphoid view is commonly obtained as part of the FAST examination, which many providers perform on a regular basis. Providers with limited POCUS experience would likely feel more confident performing this technique, compared to power Doppler assessment of the intraosseous space, as it does not require new skill acquisition. Further study into the use of this novel technique is required to support its effectiveness. 

In addition to confirming the technique’s reproducibility there are a number of factors that require investigation: How much volume is required? How fast must the fluid be administered? Does intentional agitation of the fluid enhance the technique? Does the location of the IO line have an effect? Furthermore, we discovered this technique while imaging a 4-week-old child. Further examination would want to establish the effectiveness of this technique on patients of different ages, lengths, and weights. If further study supports its effectiveness, this technique could be incorporated into the clinical assessment of IO lines in the future. 

## Conflict of Interest

The authors have no conflict of interest to declare.

## Funding

The authors received no funding.

## Patient consent

The authors gained consent from the patient to publish.

## Supplementary Material

Video S1 IVC view demonstrating a collapsible IVC.

Video S2 Subxiphoid short axis view of the heart demonstrating bubbles (hyperechoic circles) moving within the right ventricle (RV).

Video S3 Subxiphoid short axis view of the heart demonstrating bubble (hyperechoic circles) moving within the right ventricle (RV).

## References

[R183509127648779] Sampson C S (2019). Extravasation from a Misplaced Intraosseous Catheter. Clin Pract Cases Emerg Med.

[R183509127648773] Galpin R D, Kronick J B, Willis R B, Frewen T C (1991). Bilateral lower extremity compartment syndromes secondary to intraosseous fluid resuscitation. J Pediatr Orthop.

[R183509127648776] Blumberg S M, Gorn M, Crain E F (2008). Intraosseous infusion: a review of methods and novel devices. Pediatr Emerg Care.

[R183509127648778] Saul T, Siadecki S D, Berkowitz R, Rose G, Matilsky D (2015). The accuracy of sonographic confirmation of intraosseous line placement vs physical examination and syringe aspiration. Am J Emerg Med.

[R183509127648777] Stone M B, Teismann N A, Wang R (2007). Ultrasonographic confirmation of intraosseous needle placement in an adult unembalmed cadaver model. Ann Emerg Med.

[R183509127648774] Kyle A I, Auten J D, Zarow G J (2022). Determining Intraosseous Needle Placement Using Point-of-Care Ultrasound in a Swine (Sus scrofa) Model. Mil Med.

[R183509127648775] Upadhyay J, Basu S, Srivastava Y (2021). Agitated saline contrast to delineate central venous catheter position in neonates. J Perinatol.

